# Prevalence of attention-deficit/hyperactivity disorder symptoms and their associations with sleep schedules and sleep-related problems among preschoolers in mainland China

**DOI:** 10.1186/s12887-018-1022-1

**Published:** 2018-02-19

**Authors:** Hui Cao, Shuangqin Yan, Chunli Gu, Sumei Wang, Lingling Ni, Huihui Tao, Ting Shao, Yeqing Xu, Fangbiao Tao

**Affiliations:** 1 Ma’anshan Maternity and Child Health Care Hospital, Ma’anshan, Anhui 243000 China; 2 Department of Maternal, Child and Adolescent Health, School of Public Health, Anhui Medical University, 81 Mei Shan Road, Hefei, Anhui 230032 China; 3 Anhui Provincial Key Laboratory of Population Health and Aristogenics, Hefei, 230032 China

**Keywords:** Sleep, Sleep-related problems, Inattention, Hyperactivity/impulsivity, Preschooler

## Abstract

**Background:**

Attention-deficit/hyperactivity disorder (ADHD) among children is an increasing public health concern. The identification of behavioral risk factors, including sleep quality, has important public health implications for prioritizing behavioral intervention strategies for ADHD. Herein, this study aimed to investigate the prevalence of high levels of ADHD symptoms and to explore the association between sleep schedules, sleep-related problems and ADHD symptoms among preschoolers aged 3 to 6 years in mainland China.

**Methods:**

A cross-sectional study was conducted, comprising a large sample of 15,291 preschoolers in Ma’anshan city of Anhui Province in China. ADHD symptoms were assessed by the 10-item Chinese version of the Conners Abbreviated Symptom Questionnaire (C-ASQ). Sleep-related variables included caregivers’ responses to specific questions addressing children’s daytime and nighttime sleep schedules, as well as sleep-related behaviors. Data on other factors were also collected, such as socio-demographic characteristics, TV viewing duration on weekdays and weekends, and outdoor activities. Logistic regression models were used to analyze the relationships between sleep schedules, sleep-related problems and ADHD symptoms.

**Results:**

Approximately 8.6% of the total sample of preschoolers had high levels of ADHD symptoms, with boys having higher levels than girls (9.9% vs. 7.2%). In the logistic regression analysis, after adjusting for TV viewing duration, outdoor activities, and socio-demographic characteristics, delayed bedtime was significantly associated with a risk of high levels of ADHD symptoms, with odds ratios (*OR*) of 2.50 [95% confidence interval (*CI*): 2.09 ~ 3.00] and 2.04 (95% *CI*: 1.72 ~ 2.42) for weekdays and weekends, respectively. Longer time falling asleep (≥ 31 min) (*OR* = 1.76, 95% *CI*: 1.47 ~ 2.11), no naps (*OR* = 1.57, 95% *CI*: 1.34 ~ 1.84) and frequent sleep-related problems (*OR* = 4.57, 95% *CI*: 3.86 ~ 5.41) were also significantly associated with an increased risk of high levels of ADHD symptoms, while longer sleep duration (> 8.5 h) was associated with a decreased risk of high levels of ADHD symptoms (*OR* = 0.76, 95% *CI*: 0.67~ 0.87).

**Conclusions:**

ADHD symptoms are prevalent in preschoolers in Ma’anshan region, China. Undesirable sleep schedules and sleep-related problems among preschoolers confer a risk of ADHD symptoms, highlighting the finding that beneficial and regular sleep habits potentially attenuate ADHD symptoms among preschoolers.

## Background

Attention-deficit/hyperactivity disorder (ADHD) among children is a rising public health concern and a common psychiatric disorder with a childhood onset, defined by age-inappropriate symptoms of inattention and/or hyperactivity and impassivity [1]. It is well known that ADHD is associated with psychiatric and developmental disorders. The potential mechanism of ADHD is still under study. It has been associated with a broad range of negative outcomes for affected subjects [2] and with a considerable financial burden [3]. The worldwide pooled prevalence of ADHD has been estimated to be 5.29% (95% *CI*: 5.01 ~ 5.56), as noted in an extensive literature review of relevant articles from North America, South America, Europe, Africa, Asia, Oceania, and the Middle East [4]. Few population-based epidemiological studies on ADHD symptoms have been conducted in China. One such study found that the weighted 3-month prevalence rates across 3 consecutive years of ADHD symptoms (from the seventh grade to the ninth grade) were 7.5, 6.1 and 3.3% [5]. Although ADHD is most typically diagnosed during the school years, there is an increasing tendency for identification to occur among preschoolers [6]. Several longitudinal studies suggest that ADHD symptoms in preschoolers might persist through elementary school [6, 7] and into adulthood [8, 9]. Therefore, we surmised that a thorough understanding of the epidemiological features of ADHD symptoms in preschool children is important for preventing and managing this disorder.

Sleep has been referred to as a “window to the central nervous system”, owing to its close associations with many other neurophysiological variables. Children are highly vulnerable to sleep disruptions in early childhood, perhaps due to the complexity of the sleep process and children’s reliance on caregivers for achieving and maintaining sleep. Childhood sleep problems have been linked to a range of adverse health outcomes. Recently, Sivertsen, et al. showed that a short sleep duration (≤ 10 h) and frequent nocturnal awakenings (≥ 3 times) at 18 months of age significantly predicted both the concurrent and later incidence of emotional and behavioral problems at 5 years of age [10]. Nelson, et al. reported that sleep problems were negatively associated with performance on tasks assessing working memory and interference suppression inhibition, even after controlling for general cognitive abilities [11]. The relation between sleep and ADHD has gained renewed interest since clinicians and researchers observed that sleep-related problems and complaints are relatively common among children with ADHD. Shorter sleep duration and sleep disturbances have been found to appear early and predate the clinical diagnosis of ADHD [12]. In addition, the ADHD group had significantly higher subscales and total scores assessed by the Children’s Sleep Habits Questionnaire (CSHQ) [13]. In addition, a meta-analysis comparing sleep in children with ADHD versus sleep in controls indicated that children with ADHD had significantly higher bedtime resistance, more sleep onset difficulties, night awakenings, difficulties with morning awakenings, sleep-disordered breathing, and daytime sleepiness in subjective studies [14].

While there is a wealth of studies on sleep patterns in children with ADHD, few studies have focused on nonclinical samples of preschoolers or investigated the direct association between sleep-related phenomena and ADHD symptoms. In addition, most existing studies have been limited to Western populations. Therefore, we aimed to conduct a study to investigate the prevalence of high levels of ADHD symptoms in a nonclinical sample of Chinese preschoolers and to explore the relationships between sleep schedules, sleep-related problems and ADHD symptoms.

## Methods

### Participants

In 2014, a school-based cross-sectional survey was conducted in 91 kindergartens in Ma’anshan city of Anhui Province in China. A total of 16,439 children were recruited to participant in the study. Cases with missing values for more than 15% of items were excluded from this study. Ultimately, the data of the 15,291 preschoolers (8218 boys and 7073 girls) with complete assessments by their caregivers were used for data analysis. The participants’ ages ranged from 3 to 6 years old, with a mean age of 4.91 years (standard deviation (SD) =1.00).

### Instruments

A self-administered questionnaire containing information on socio-demographic characteristics, TV viewing duration on weekdays and weekends, outdoor activities, sleep schedules and sleep-related problems, and ADHD symptoms was completed by preschoolers’ caregivers at home and returned to the teacher the next day. Then, graduate students majoring in Maternal and Child Health recovered all questionnaires from the kindergartens one by one. To guarantee the quality of questionnaires, quality control was conducted by teachers and graduate students separately.

#### Sleep schedules and sleep-related problems

The sleep questionnaire adapted from the Children’s Sleep Habits Questionnaire (CSHQ) [15], Pittsburgh Sleep Quality Index (PSQI) [16], and Children’s sleep status questionnaire [17], included specific questions about children’s daytime and nighttime sleep schedules, as well as sleep-related behaviors.

Daytime and nighttime sleep schedules were assessed with seven questions. Four of the questions were as follows. The first and second items were “When does your child normally go to bed on a usual weekday, as well as a weekend day?” Response options were “before 7:00 PM”, “between 7:00 and 8:00 PM”, “between 8:00 and 9:00 PM”, “between 9:00 and 10:00 PM”, and “after 10:00 PM”. Because of small frequencies, “before 7:00 PM”, “between 7:00 and 8:00 PM”, “between 8:00 and 9:00 PM” were regrouped into the broader category “before 9:00 PM”. The third and fourth items were “When does your child normally wake up on a usual weekday, as well as a weekend day?” Response options were “before 6:00 AM”, “between 6:00 and 8:00 AM”, “between 8:00 and 9:00 AM”, “between 9:00 and 11:00 AM”, and “after 11:00 AM”. Because of small frequencies, “between 8:00 and 9:00 AM”, “between 9:00 and 11:00 AM” and “after 11:00 AM” were regrouped into the broader category “after 8:00 AM”. The fifth question was “How long does it take your child fall-asleep at night?” Response options were “less than 15 min”, “16 ~ 30 min”, “31 ~ 60 min” and “more than 60 min”. This study categorized falling asleep as “less than 15 min”, “16 ~ 30 min” and “more than 30 min”. The sixth question was an open question: “Indicate how long, in total, your child has slept during the night (on average) in the last month. Do not count the hours that your child is awake”. Sleep duration was classified into “less than or equal to the 25th percentile (*P*_25_) of the score (8.5 h)”, “8.6 ~ 9.4 h” and “greater than or equal to the 75th percentile (*P*_75_) of the score (9.5 h)”. The seventh item was “Is your child in the habit of having naps?” Response options were “often”, “occasionally” and “no”.

Sleep-related problems: night waking, falling sleep in the evening, bed-wetting, bruxism, sweating in sleep, mouth breathing, sleep talking, snoring, nightmares, and shouting in sleep. Each item has five answer options based on the duration of each symptom (none at all, 1 time/week, 2 times/week, 3 ~ 4 times/week, 5 or more times/week). Total score of sleep-related problems was calculated and then transformed into 3 categories (with less than or equal to *P*_25_ as no sleep-related problems, *P*_25_ to *P*_75_ as occasionally having sleep-related problems, and greater than or equal to *P*_75_ as often having sleep-related problems).

#### ADHD symptoms

The 10-item Chinese version of the Conners Abbreviated Symptom Questionnaire (C-ASQ) is derived from the Revised Conners Parent Rating Scale [18]. It assesses ADHD symptoms on a 4-point scale ranging from 0 to 3. It discriminates very well between children with and without ADHD and therefore has been used as a valid screening instrument for the identification and measurement of the behavioral problems of ADHD among children in China [19]. The total score is coded into a categorical variable, with a score ≥ 15 representing high levels of ADHD symptoms. Its sensitivity, specificity and accuracy are 76.0, 92.9 and 82.1%, respectively [20].

#### Potential confounding factors

The factors that were considered potential confounders were socio-demographic characteristics, TV viewing duration on a usual weekday and weekend, and outdoor activities. The socio-demographic characteristic variables included age (from 3 to 6 years old), gender (boy and girl), household registration (urban and rural), parental age (less than or equal to *P*_25_, between *P*_25_ and *P*_75_, and greater than or equal to *P*_75_), and self-reported monthly household income per capita (999 yuan or less, 1000 ~ 2999 yuan, and 3000 yuan or more). Education level groups were defined as illiteracy or primary school, junior high school, senior high school, junior college (i.e., 1 ~ 3 years of college), and university or above (i.e., four or more years of college). Because of small frequencies, illiteracy, primary, and junior high school were regrouped into the broader category “middle school or below”. Senior high school and junior college were regrouped into the broader category “senior high school or junior college”. Based on this recommendation the American Academy of Pediatrics, TV viewing time was classified into < 1 h/d and ≥ 1 h/d on weekdays, and < 2 h/d and ≥ 2 h/d on weekends. Frequencies of outdoor activities were reported as often, sometimes and seldom.

#### Missing values

Individuals were excluded from the analyses if she/he had more than 15% items with missing values. Otherwise, the missing value was replaced by the mean value of that particular item for that individual.

### Data analysis

Data were entered into an EpiData 3.1 database. All the data were analyzed using the Statistical Package for Social Sciences (SPSS version 13.0). Descriptive analyses were performed on all variables and the prevalence of high levels of ADHD symptoms. Pearson’s chi-square was employed to compare the proportions of the independent variables versus the dependent variables. Associations between sleep problems and ADHD symptoms were examined using logistic regression models. Odds ratios (*OR*) and their 95% confidence intervals (*CI*) were calculated. *P* values less than 0.05 were considered significant for all tests.

## Results

Table 1 represents the general characteristics of preschoolers and the relationship between ADHD symptoms and general characteristics. Approximately 8.6% (1317) were identified as having high levels of ADHD symptoms, and boys comprised 61.6% of this group. The rate of high levels of ADHD symptoms decreased with increasing age (*χ*^*2*^ *=* 32.8*, P*< 0.001). More boys had high levels of ADHD symptoms than girls (9.9% vs. 7.2%, *χ*^*2*^ *=* 35.59, *P*< 0.001). Preschoolers with particular factors were more likely to have high levels of ADHD symptoms, such as rural household registration, lower parental age (less than or equal to 28 years), lower parental education level (middle school or below), TV viewing time ≥ 1 h/d on weekday and ≥ 2 h/d on weekend, and seldom participating in outdoor activities.

**Table 1:** Sample characteristics of high ADHD symptoms among preschoolers

Variables	Total	High ADHD symptoms [n (%)]	*χ* ^*2*^	*P*
Age				
3 year	3396	344 (10.1)	32.80	< 0.001
4 year	4525	436 (9.6)		
5 year	4442	331 (7.5)		
6 year	2928	206 (7.0)		
Gender				
Male	8218	811 (9.9)	35.59	< 0.001
Female	7073	506 (7.2)		
Only child				
Yes	11,402	1005 (8.8)	2.31	0.129
No	3889	312 (8.0)		
Household registration				
Rural	5857	582 (9.9)	21.14	< 0.001
Urban	9434	735 (7.8)		
Maternal age				
≤ *P*_25_ (28 year)	3867	459 (11.9)	71.12	< 0.001
*P*_25_~ *P*_75_ (29~ 34 year)	7083	549 (7.8)		
≥ *P*_75_ (34 year)	4341	309 (7.1)		
Maternal education				
Middle school or below	6485	684 (10.5)	73.76	< 0.001
Senior high school or junior college	6480	518 (8.0)		
University or above	2326	115 (4.9)		
Paternal age				
≤ *P*_25_ (31 year)	4592	506 (11.0)	48.38	< 0.001
*P*_25_~ *P*_75_(31~ 37 year)	6616	497 (7.5)		
≥ *P*_75_(37 year)	4083	314 (7.7)		
Paternal education				
Middle school or below	5253	538 (10.2)	44.33	< 0.001
Senior high school or junior college	7207	612 (8.5)		
University or above	2831	167 (5.9)		
Monthly household income per capita(yuan/RMB)				
999 or less	744	61 (8.2)	0.71	0.701
1000~ 2999	7047	596 (8.5)		
3000 or more	7500	660 (8.8)		
TV viewing time on weekday				
<1 h/d	4089	262 (6.4)	34.49	< 0.001
≥ 1 h/d	11,202	1055 (9.4)		
TV viewing time on weekend				
<2 h/d	5395	352 (6.5)	46.19	< 0.001
≥ 2 h/d	9896	965 (9.8)		
Outdoor activities				
Often	8210	672 (8.2)	16.52	< 0.001
Sometimes	5787	495 (8.6)		
Seldom	1294	150 (11.6)		

Table 2 shows the rates of high levels of ADHD symptoms among preschoolers with different sleep schedules and sleep-related problems. The bedtimes on weekdays and weekends, waking times on weekends, time falling asleep, sleep duration, naps and sleep-related problems were significantly associated with ADHD symptoms in *χ*^*2*^ tests (all *P*< 0.05). Preschoolers more likely to have high levels of ADHD symptoms were those who had delayed bedtimes, longer time falling asleep, shorter sleep duration, no naps, and frequent sleep-related problems.

**Table 2:** The prevalence of high ADHD symptoms among preschoolers with different sleep schedules and sleep-related problems

Sleep schedules and sleep-related problems	Total	High ADHD symptoms [n (%)]	*χ* ^*2*^	*P*
Bedtime on weekday				
Before 9:00 PM	5828	417 (7.2)	90.54	< 0.001
Between 9:00 and 10:00 PM	8032	685 (8.5)		
After 10:00 PM	1431	215 (15.0)		
Bedtime on weekend				
Before 9:00 PM	3673	256 (7.0)	53.49	< 0.001
Between 9:00 and 10:00 PM	8300	675 (8.1)		
After 10:00 PM	3318	386 (11.6)		
Waking time on weekday				
Before 6:00 AM	629	61 (9.7)	1.66	0.437
Between 6:00 and 8:00 AM	14,249	1216 (8.5)		
After 8:00 AM	413	40 (9.7)		
Waking time on weekend				
Before 6:00 AM	361	39 (10.8)	9.85	0.007
Between 6:00 and 8:00 AM	9640	780 (8.1)		
After 8:00 AM	5290	498 (9.4)		
Time falling asleep				
≤ 15 min	2813	187 (6.6)	70.31	< 0.001
16~ 30 min	8561	669 (7.8)		
≥ 31 min	3917	461 (11.8)		
Sleep duration				
≤ 8.5 h	4570	467 (10.2)	21.36	< 0.001
8.6~ 9.4 h	6458	512 (7.9)		
≥ 9.5 h	4263	338 (7.9)		
Having naps				
Often	9437	728 (7.7)	33.19	< 0.001
Occasionally	3737	347 (9.3)		
No	2117	242 (11.4)		
Having sleep-related problems				
Often	4017	639 (15.9)	408.79	< 0.001
Occasionally	6508	485 (7.5)		
No	4766	193 (4.0)		

As shown in Table 3, in the logistic regression analysis, after adjusting for TV viewing duration, outdoor activities, and socio-demographic characteristics, delayed bedtime was significantly associated with a risk of high levels of ADHD symptoms, with *ORs* of 2.50 (95% *CI*: 2.09 ~ 3.00) and 2.04 (95% *CI*: 1.72 ~ 2.42) for weekdays and weekends, respectively. Longer time falling asleep (≥31 min) (*OR* = 1.76, 95% *CI*: 1.47 ~ 2.11), no naps (*OR* = 1.57, 95% *CI*: 1.34 ~ 1.84) and frequent sleep-related problems (*OR* = 4.57, 95% *CI*: 3.86 ~ 5.41) were also significantly associated with an increased risk of high levels of ADHD symptoms, while a longer sleep duration (> 8.5 h) was associated with a decreased risk of high levels of ADHD symptoms (*OR* = 0.76, 95% *CI*: 0.67 ~ 0.87).

**Table 3:** Crude and Adjusted odds ratios for the relationships between sleep and ADHD symptoms among preschoolers

Sleep schedules and sleep-related problems	High ADHD symptoms			
	Crude *OR* (95% *CI*)	*P*	Adjusted^a^*OR *(95% *CI*)	*P*
Bedtime on weekday				
Before 9:00 PM	1.00		1.00	
Between 9:00 and 10:00 PM	1.21 (1.06 ~ 1.37)	0.003	1.36 (1.19 ~ 1.54)	< 0.001
After 10:00 PM	2.29 (1.92 ~ 2.74)	<0.001	2.50 (2.09 ~ 3.00)	< 0.001
Bedtime on weekend				
Before 9:00 PM	1.00		1.00	
Between 9:00 and 10:00 PM	1.18 (1.02 ~ 1.37)	0.029	1.36 (1.17 ~ 1.58)	< 0.001
After 10:00 PM	1.76 (1.49 ~ 2.07)	<0.001	2.04 (1.72 ~ 2.42)	<0.001
Time falling asleep				
≤ 15 min	1.00		1.00	
16~ 30 min	1.19 (1.01 ~ 1.41)	0.042	1.15 (0.97 ~ 1.37)	0.097
≥ 31 min	1.87 (1.56 ~ 2.24)	<0.001	1.76 (1.47 ~ 2.11)	< 0.001
Sleep duration				
≤ 8.5 h	1.00		1.00	
8.6~ 9.4 h	0.76 (0.66 ~ 0.86)	<0.001	0.76 (0.67 ~ 0.87)	< 0.001
≥ 9.5 h	0.76 (0.65 ~ 0.88)	<0.001	0.72 (0.62 ~ 0.84)	< 0.001
Having naps				
Often	1.00		1.00	
Occasionally	1.23 (1.07 ~ 1.40)	0.003	1.19 (1.04 ~ 1.36)	0.013
No	1.54 (1.32 ~ 1.80)	<0.001	1.57 (1.34 ~ 1.84)	< 0.001
Having sleep-related problems				
No	1.00		1.00	
Occasionally	1.91 (1.60~ 2.26)	<0.001	1.91 (1.60~ 2.26)	<0.001
Often	4.48 (3.79~ 5.30)	<0.001	4.57 (3.86~ 5.41)	<0.001

^a^Adjusted for age, gender, household registration, parental age and education level, self-reported monthly household income per capita, TV viewing on weekday and weekend, outdoor activities

## Discussion

The information provided here may help us to understand the prevalence of high levels of ADHD symptoms and the relationships between ADHD symptoms and sleep schedules, sleep-related problems among Chinese preschoolers. This study showed that 8.6% of the total sample of preschoolers had high levels of ADHD symptoms, including 10.1% for 3-year-olds, 9.6% for 4-year-olds, 7.5% for 5-year-olds and 7.0% for 6-year-olds, with a higher rate in boys (9.9%) than girls (7.2%). The prevalence of ADHD-related behavior, or ADHD symptoms, has been estimated by epidemiological studies to range widely, from 2 to 18% in Western countries, and the prevalence of ADHD appears to be increasing in these areas [21]. The rate of ADHD symptoms in this study is lower than that reported in the study by Hebrani, et al., which revealed that the prevalence of ADHD in preschool-aged children in northeast Iran was 12.3% (95% *CI*: 10.3 ~ 14.2%) [22]. However, the prevalence varies due to a number of factors, including various diagnostic criteria, the age and gender of the population, socioeconomic status, and residence. In Arab countries, the prevalence of total ADHD symptoms, hyperactive-type symptoms and inattention-type symptoms ranged between 1.3 ~ 16%, 1.4 ~ 7.8%, and 2.1 ~ 2.7%, respectively [23]. In Iran, among 1403 children aged 3 ~ 6 years, 362 (25.8%) and 239 (17%) were classified as having ADHD symptoms according to parents’ and teachers’ reports, respectively [24]. In Japan, one study indicated that 91 (15.6%) of the 583 children selected were considered to possibly have ADHD [25]. A US-based study reported a significant increase in the parent-reported prevalence of ADHD among 3 ~ 10-year-old children during 1997–2008 [26]. Previous studies have identified gender, parental education and television watching as risk factors for ADHD symptoms [24, 27], which were findings similar to our results. The prevalence of high levels of ADHD symptoms among preschoolers and its potential long-term consequences make it an important topic to study in relation to risk and protective factors; in addition, these findings strongly imply the need to identify strategies to reduce this problem.

In our nonclinical sample, we found a clear relationship between undesirable sleep schedules (such as delayed bedtimes, longer time falling asleep, shorter sleep duration and no naps), sleep-related problems and ADHD symptoms among preschoolers from a large citywide database in China, which is consistent with findings from previous studies in clinical samples of children with ADHD [28, 29]. A large population study in 10,596 Australian preschool children found that compared to children with mild sleep problems, children with moderate/severe sleep problems (difficulties in initiating and maintaining sleep, snoring and tiredness in the mornings) were 12.1 times more likely to have diagnoses of attention-deficit disorder [28]. Another study documented that compared to the control group (community children), children with ICD-10 hyperkinetic disorder showed significantly delayed bedtimes, stronger bedtime resistance, longer sleep latency, shorter sleep duration, more frequent behavior problems and symptoms such as falling asleep in parents’ beds, needing something special to initiate sleep, having nightmares, sleep talking, experiencing sleep bruxism, having a fear of darkness, bed-wetting, and, most notably, loud snoring [29]. A meta-analysis by Cortese, et al. addressed this question by examining 16 studies of children and adolescents with ADHD who were not medicated. Children with ADHD were more likely to have bedtime resistance, sleep onset difficulties, night awakenings, difficulties with morning awakenings, sleep-disordered breathing, and daytime sleepiness than non-ADHD controls, according to parent reports [14]. Another recent meta-analysis of relevant polysomnographic studies revealed that ADHD symptoms are related to sleep-disordered breathing [30].

Delayed bedtime was significantly associated with high levels of ADHD symptoms after adjusting for TV viewing duration on weekdays and weekends, outdoor activities and socio-demographic factors. This finding also corresponds to analogous investigations of sleep schedules [31–33]. Kobayashi, et al. found that in comparison to an early bedtime, the *OR* of an irregular or late bedtime at 2 years of age, with the outcome of attention problems at 8 years of age, was 1.62 (95% *CI*: 1.12 ~ 2.36) [31]. Other studies have also reported that short sleep duration was associated with ADHD-like symptoms of inattention [32, 33]. Experimental research has consistently demonstrated that napping during the daytime improves cognitive functioning [34], psychomotor performance, memory and even mood [35]. Although these effects are well established, it remains relatively unknown whether routine napping is common in preschoolers who are likely to benefit from improvements in ADHD symptoms. This study showed that having no naps (*OR* = 1.57, 95% *CI*: 1.34 ~ 1.84) was significantly associated with high levels of ADHD symptoms. Similar to the findings of the previous study, a higher frequency of daily napping was associated with less prosocial behavior and a reduced ability to address challenges, whereas difficulty in settling for naps was also associated with poorer behavior and adjustment in preschool [36]. In addition, outcomes of a systematic review showed that the evidence regarding behavior is less certain. More systematic and well-designed studies are needed [37].

Poor sleep has deleterious effects on the development of brain functions [38]. It has been suggested that an irregular lifestyle and late hours for waking and bedtimes can cause disturbances in various aspects of biological rhythms [39]. ADHD is thought to reflect dysfunctions not only in the prefrontal-striatal circuitry but also in large-scale resting-state neural networks [40]. The mechanisms explaining the relationship between sleep and ADHD are complex, and there are several potential etiologies that are not mutually exclusive. As noted by Owens [41] sleep problems may mimic ADHD symptomatology, exacerbate underlying ADHD symptoms, and be associated with or be exacerbated by ADHD, and the psychotropic medications used to treat ADHD may result in sleep problems.

### Strengths and limitations

Some strengths and limitations should be noted. First, this study was cross-sectional, which did not allow for causality or the direction of relationships to be determined. However, the appropriate analysis of cross-sectional data represents a useful initial step in identifying associations between sleep schedules, sleep-related problems and ADHD symptoms. Second, the study subjects were sampled within Ma’anshan city. It is appropriate to assume that the sampled population represents the preschoolers in the city of Ma’anshan, but it is far from being a good sample at the provincial or national level. Third, our study incorporated only the subjective measures obtained from caregiver reports. Further studies are needed that utilize objective sleep measures for assessing the relationship between sleep physiopathology and ADHD symptoms in Chinese preschoolers.

Despite these limitations, this study investigated the prevalence of high levels of ADHD symptoms and examined the associations between sleep schedules, sleep-related problems and ADHD symptoms based on data from a citywide representative sample of preschoolers in mainland China. The findings suggest that high levels of ADHD symptoms were fairly common among Chinese preschoolers and underpin the importance of understanding the relationship between sleep schedules, sleep-related problems and ADHD symptoms. Future studies may be warranted to investigate whether improvement in sleep schedules and sleep-related problems in preschoolers can alleviate ADHD at a later age. The health department of the Chinese government has now recognized high levels of ADHD symptoms as a serious public health problem. Arguably, it is time for the World Health Organization and health departments around the world to develop effective health policies to increase public awareness of high levels of ADHD symptoms.

## Conclusions

In conclusion, nearly 1 in 10 children aged 3 ~ 6 years old have high levels of ADHD symptoms in China. Given the significant association between undesirable sleep schedules, sleep-related problems and ADHD symptoms among preschoolers, our results highlight that beneficial and regular sleep habits potentially attenuate ADHD symptoms among preschoolers. It might be meaningful for child health practitioners to include these findings regarding sleep schedules and related problems in their assessments of children with ADHD-related symptoms.

## Abbreviations

*χ*^2^: Chi square
ADHD: Attention deficit and hyperactivity disorder
C-ASQ: Conners’ abbreviated symptom questionnaire
CI: Confidence interval
CSHQ: Children’s sleep habits questionnaire
OR: Odds ratio
